# Resilience and Brain Changes in Long‐Term Ayahuasca Users: Insights From Psychometric and fMRI Pattern Recognition

**DOI:** 10.1002/jmri.70063

**Published:** 2025-08-20

**Authors:** Lucas Rego Ramos, Orlando Fernandes, Tiago Arruda Sanchez

**Affiliations:** ^1^ Programa de Pós‐graduação em Neurociência Translacional – PGNET Universidade Federal do Rio de Janeiro Rio de Janeiro Brazil; ^2^ Programa de Pós‐graduação em Anatomia Patológica Universidade Federal do Rio de Janeiro Rio de Janeiro Brazil; ^3^ Laboratório de Neuroimagem e Psicofisiologia – Programa de Pós‐graduação em Saúde Mental e Psiquiatria Instituto de Psiquiatria – Universidade Federal do Rio de Janeiro Rio de Janeiro Brazil; ^4^ Laboratório de Neurofisiologia e Comportamento – Departamento de Fisiologia e Farmacologia Instituto Biomédico – Universidade Federal Fluminense Nitéroi Brazil

**Keywords:** Ayahuasca, emotion regulation, functional magnetic resonance imaging (fMRI), machine learning, pattern recognition

## Abstract

**Background:**

Ayahuasca is an Amazonian psychedelic brew that contains dimethyltryptamine (DMT) and beta carbolines. Prolonged use has shown changes in cognitive‐behavioral tasks, and in humans, there is evidence of changes in cortical thickness and an increase in neuroplasticity factors that could lead to modifications in functional neural circuits.

**Purpose:**

To investigate the long‐term effects of Ayahuasca usage through psychometric scales and fMRI data related to emotional processing using artificial intelligence tools.

**Study Type:**

Retrospective Cross‐sectional, case–control study.

**Subjects:**

38 healthy male participants (19 long‐term Ayahuasca users and 19 non‐user controls).

**Field Strength/Sequence:**

1.5 Tesla; gradient‐echo T2*‐weighted echo‐planar imaging sequence during an implicit emotion processing task.

**Assessment:**

Participants completed standardized psychometric scales including the Ego Resilience Scale (ER89). During fMRI, participants performed a gender judgment task using faces with neutral or aversive (disgust/fear) expressions. Whole‐brain fMRI data were analyzed using multivariate pattern recognition.

**Statistical Tests:**

Group comparisons of psychometric scores were performed using Student's t‐tests or Mann–Whitney U tests based on normality. Multivariate pattern classification and regression were performed using machine learning algorithms: Multiple Kernel Learning (MKL), Support Vector Machine (SVM), and Gaussian Process Classification/Regression (GPC/GPR), with *k*‐fold cross‐validation and permutation testing (*n* = 100–1000) to assess model significance (*α* = 0.05).

**Results:**

Ayahuasca users (mean = 43.89; SD = 5.64) showed significantly higher resilience scores compared to controls (mean = 39.05; SD = 5.34). The MKL classifier distinguished users from controls with 75% accuracy (*p* = 0.005). The GPR model significantly predicted individual resilience scores (*r* = 0.69).

**Data Conclusion:**

Long‐term Ayahuasca use may be associated with altered emotional brain reactivity and increased psychological resilience. These findings support a neural patterns consistent with long‐term adaptations of Ayahuasca detectable via fMRI and machine learning‐based pattern analysis.

**Evidence Level:**

4.

**Technical Efficacy:**

Stage 1.

## 1 Plain Language Summary

This study explored how long‐term use of Ayahuasca, a traditional Amazonian psychedelic, may affect emotional resilience and brain activity. We compared 19 regular Ayahuasca users with 19 non‐users using psychological questionnaires and brain scans while participants viewed emotional images. We found that Ayahuasca users had higher resilience scores and showed distinct patterns of brain activity related to emotion processing. Using artificial intelligence, we could predict resilience levels based on brain patterns. These results suggest that long‐term Ayahuasca use may be linked to lasting changes in emotional regulation and brain function, potentially supporting psychological well‐being.

## 2 Introduction

The use of psychoactive plants is part of the culture in several civilizations around the world. Ayahuasca is a psychedelic substance in expansion in Brazil and the world, mostly used for religious purposes in traditional indigenous rituals and other ritualistic syncretic practices. Ayahuasca is made from the decoction of two plants, Psychotria viridis and *Banisteriopsis caapi* . Psychotria viridis contains DMT (*N,N*‐Dimethyltryptamine) [1], while *B. caapi* contains beta‐carboline alkaloids such as harmine, harmaline, and tetrahydroharmine. These components promote a synergistic action in the serotonergic system: DMT acts as an agonist at the 5‐HT2A receptor, while the alkaloids function as a monoamine oxidase inhibitor (i‐MAO)—enzymes that would otherwise rapidly degrade DMT. Recently, a scientific study has focused on clinical trials and experimental studies to evaluate its applications in psychiatric disorders and to investigate which psychophysiological mechanisms are most involved [2].

Ayahuasca ingestion promotes changes in brain biochemistry and induces changes in perception, emotion, and cognitive processes [3]. Almeida et al. performed the first randomized, double‐blind, placebo‐controlled clinical trial exploring the modulation of brain‐derived neurotrophic factor (BDNF) in response to Ayahuasca ingestion in patients with depression and controls, and an increase in BDNF was observed in volunteers who ingested Ayahuasca [4]. BDNF is a protein with an important role in neural survival and neurogenesis [4]. Ayahuasca also enhances the expression of sigma receptor 1, a versatile, stress‐responsive receptor known to support cell survival, neuroprotection, neuroplasticity, and neuromodulation [4]. Psychophysiological changes in healthy subjects were shown due to acute use of Ayahuasca in measures of the emotional dysregulation scale [5], an increase in somatic symptoms, affect, volition, cognition, perception, stimulatory effects, positive mood, and euphoria [6, 7]. The acute effect of Ayahuasca promotes changes in functional connectivity in the default mode network (DMN) [8] and in blood volume in emotional processing regions, such as the amygdala, insula, anterior cingulate, and frontal regions [6, 7]. A previous study showed a decrease in amygdala activity in an implicit emotional stimulus after the acute use of Ayahuasca [9].

Machine learning techniques have increasingly been used to uncover how substances affect brain function. For example, in patients with major depressive disorder undergoing psilocybin treatment, machine learning models revealed that specific connectivity patterns—particularly between frontal, temporal, and occipital regions—can predict both the immediate and sustained reduction of depressive symptoms, and fronto‐temporal connectivity was able to predict both initial and delayed reductions in depressive symptoms [10]. Rather than merely identifying abstract patterns in neuroimaging data, these approaches help isolate the neural signatures that underlie the pharmacological effects of the drug. Similarly, models predicting brain age based on gray matter morphology in long‐term cannabis users [11] suggest drug‐induced neurobiological changes that may not be captured through conventional analyses. In another study, Bedford et al. [12] applied machine learning to functional connectivity data from LSD trials, successfully distinguishing the drug condition from placebo with high accuracy. These findings illustrate how machine learning enables a more precise characterization of the brain's functional response to psychoactive compounds, providing insights into mechanisms of action that traditional statistical methods may overlook.

Only a limited number of studies have applied multivariate neuroimaging analysis methods to investigate the effects of psychedelic substances on brain function or to predict individual psychometric traits [10, 12] Thus, the aim of the present study is to use these approaches to evaluate differences in brain activity between experienced Ayahuasca users, without the influence of the substance, and control participants during emotional tasks. The hypothesis to be tested is that the long‐term action of the alkaloid components of Ayahuasca, mainly in the serotonergic system, interferes with the processing of emotions beyond the acute phase, modulating brain responses in limbic and prefrontal structures. Therefore, the present study aims to evaluate the long‐term effect of Ayahuasca use on emotional brain processing and psychological resilience.

## 3 Materials and Methods

### 3.1 Sample Characteristics

Twenty‐three male experienced users of Ayahuasca were selected among members of two Ayahuasca communities in Rio de Janeiro—Brazil. Both communities are in urban areas, although their ritual structures differ slightly in format and duration. Nineteen male nonusers were recruited from local advertisements at the Universidade Federal do Rio de Janeiro (UFRJ). This study was previously approved by the Ethics Committee of Hospital Universitário Clementino Fraga Filho (HUCFF) at UFRJ (CAEE: 40806315.2.0000.5257). All participants were informed about the objectives of the study protocol, and they gave their written consent.

For all participants, exclusion criteria included: (a) history of psychiatric or neurological diseases; (b) use of medications or substances that affect the nervous or vascular system; (c) MRI contraindications, such as claustrophobia or metal parts in the body like cardiac pacemakers, stents, implants, and so on; (d) having gone through a situation of great stress recently at the time of the exam. In addition to the preceding criteria, participants in the Ayahuasca group would be excluded for ingesting Ayahuasca within 1 week of the fMRI exam. Inclusion criteria for all participants comprised the following: (a) male; (b) age greater than 18 years. The inclusion criteria for the Ayahuasca group were to have lifetime experience with Ayahuasca at least 36 times in a one‐year period. This criterion was based on ethnographic data from Brazilian urban religious communities, where weekly Ayahuasca ceremonies are common. All participants were lay members of these communities and not spiritual leaders, which helps minimize confounding factors related to leadership roles or responsibilities.

Forty‐two subjects were initially recruited for two different groups, with 23 in the Ayahuasca group and 19 in the nonusers' group. A total of four of these participants were excluded from the Ayahuasca group due to: (1) the presence of metallic dental braces; (1) incorrect performance of the task; (1) withdrawal; (1) excessive movement during the exam (more than 2 mm or 2°). The final sample was composed of 38 participants. Both groups were comprised of 19 participants, with the mean age of experienced users being 31.5 (SD = 10.7; range: 23–68) and the mean age of the nonuser being 33.1 years (SD = 12.5; range: 20–58) (*p* = 0.68). Participants in the Ayahuasca group reported a mean of 8.3 years of regular use and had ingested the substance at least 36 times within a one‐year period.

### 3.2 Psychometric Assessments

The volunteers completed seven self‐assessment psychometric scales before the fMRI exam, namely: (i) Positive and Negative Affect Schedule—PANAS translated and adapted for Brazil [13]; (ii) Eco‐Resiliency Scale—ER89 [14]; (iii) Beck's Anxiety Inventory—BAI translated and adapted for Brazil [15]; (iv) Beck's Depression Inventory—BDI translated and adapted for Brazil [16]; (v) State–Trait Anxiety Inventory—STAI translated and adapted for Brazil [17].

Assessment of subjects' mood trait was done by self‐report in PANAS, describing two dominant dimensions: positive affective and negative affective. PANAS is a 20‐item scale consisting of 10 adjectives that describe positive mood (active; alert; attentive; determined; enthusiastic; excited; inspired; interested; proud; strong) and another 10 words related to negative moods (distresses; upset; hostile; irritable; scared; afraid; ashamed; guilty; nervous; jittery), rating the degree to which they experienced each emotion on a 1–5 scale (1 = very slightly or not at all; 5 = extremely).

The ER89 resilience scale contains 14 items answered on an ordinal scale (1 = does not apply at all; 2 = applies slightly, if at all; 3 = applies somewhat; 4 = applies very strongly) which seeks to assess the individual psychological resilience trait, that is the ability to adapt to the misfortunes of adulthood. The higher score on this scale represents greater resilience of the subject.

Mental health symptoms were investigated using the Beck Anxiety Inventory and the Beck Depression Inventory. The Beck Anxiety Inventory consists of 21 items describing common symptoms of anxiety, being graded on a 4‐point scale, ranging from 0 (not at all) to 3 (severely). The total score can vary from 0 to 63 and can be categorized as 0–10 (minimum); 11–19 (mild); 20–30 (moderate); and 31–63 (severe). The Beck Depression Inventory evaluated the presence of symptoms of depression. This instrument is composed of 21 categories of symptoms and attitudes, which describe behavioral, cognitive, affective, and somatic manifestations. The final classification is stratified as absence of depression or minimal depressive symptoms (up to 9 points); mild‐to‐moderate depression (10–18 points); moderate depression (19–29 points); and severe depression (30–63 points).

The STAI is an instrument used to quantify subjective components related to anxiety. According to this inventory, the state scale reflects a transient reaction directly related to a situation of adversity that presents itself at a given moment, so requires the subject to describe how he feels “feel right now, that is, at this moment” in relation to 20 items presented on a 4‐point Likert scale (1 = not at all; 2 = somewhat; 3 = moderately so; 4 = very much so). The trait scale refers to a more stable aspect related to the individual's propensity to deal with greater or lesser anxiety throughout his life. The trait scale is also made up of 20 items, but the subject receives the instruction that he must respond as “generally feel” according to a new 4‐point Likert scale (1 = almost never; 2 = sometimes; 3 = often; 4 = almost always). One of the subjects in the control group did not complete the STAI questioner.

### 3.3 Imaging Experimental Design

We conducted a cross‐sectional study using an fMRI brain mapping protocol to evaluate patterns of brain activation related to emotional reactivity in experienced Ayahuasca users, compared to nonusers, as a long‐term effect. An implicit emotion processing task was presented to participants, and they were instructed to identify whether the faces were male or female [18], through a device containing two buttons, where the left button corresponded to female faces and the right button to male faces. The task stimulus included emotional expressions including neutral or aversive emotional faces (disgust and fear). They were neither asked to examine the facial expression nor to decide on the emotions expressed.

The experiment was separated into two runs and composed of a block design. Each run consisted of 15 blocks and was composed of 7 blocks of aversive faces (A) containing 8 different images and 8 blocks of neutral faces (*n*), with a duration of 17 s for each block. The sequence of image presentation was nAnAnAnAnAnAnAn, for a total of 4.3 min per run. The entire MRI scan lasted 20 min, including the extra time for structural high‐resolution T1‐weighted images (8 min) plus time for positioning and scan preparation. All faces were selected from the Facial Affect Series [19] and the stimulus protocol was created through E‐Prime software (Psychology Software Tools, E‐Studio 2.0.10.242). Each face was presented for 2 s, regardless of the participants' response, followed by a 0.125 s fixation cross.

The implicit emotion processing task was performed by subjects during an fMRI scanning in a 1.5 Tesla MRI (Magnetom Avanto; Siemens Medical System, Erlangen, Germany). The task presentation was synchronized with the fMRI acquisition using a triggering circuitry. The subjects' head movements were restrained with foam padding. Brain functional images were acquired using a gradient‐echo planar imaging sequence T2* (repetition time (TR) = 2000 ms; echo time (TE) = 40 ms; Field of View (FOV) = 256 mmm; 64 × 64 matrix; flip angle = 90°; slice thickness = 4 mm; acquisitions = 129 scans; number of slices = 25; and interleaved acquisition method) and brain high‐resolution structural images were acquired using a T1‐weighted image (TR = 2730 ms; TE = 3.26 ms; flip angle = 7°; slice thickness = 1.33; number of slices = 128; and 256 × 256 matrix, FOV = 256 mm).

### 3.4 Imaging Analysis

The hemodynamic responses according to the blood oxygenation level‐dependent (BOLD) fMRI signal changes were analyzed using SPM12 (Statistical Parametric Mapping, version 12) by general linear model (GLM) approach analysis. The data set was corrected for motion and slice time correction, and it was filtered in the space domain (10 mm FWHM—Full Width Half Maximum) and filtered in the time domain (high‐pass filter at 0.01 Hz). Individual function maps were normalized into the MNI anatomical space. After preprocessing, first‐level analysis was performed on each subject using the GLM with a boxcar waveform convolved with a canonical hemodynamic response function. One regressor of interest was created for both aversive stimuli (fearful and disgust faces) that correspond to the main experimental condition. Movement parameters were also entered as covariates to control participants' movement. We used a custom‐created mask to exclude voxels common to all images that had “NaN” (not a number). This mask can improve the performance of pattern recognition analyses by decreasing the number of non‐informative features and voxels in the model [20].

### 3.5 Machine Learning Analysis

The machine learning analysis was performed in PRoNTo (Pattern Recognition for Neuroimaging Toolbox) [21], using Matlab 2016b. In the pattern recognition analysis in favor of group discrimination, the Multiple Kernel Learning (MKL) algorithm [22]. It is noteworthy that in this model, it can lead to a better generalization performance when the clustering structure imposed by the atlas is consistent with the data, and it can identify a subset of brain regions relevant to the predictive model [23]. The MKL algorithm used all fMRI voxels and empirical knowledge of brain spatial segmentation (Automated Anatomical Labeling v.3, AAL3 atlas) [24] to discriminate between those who belong to the group of experienced Ayahuasca users and those who belong to the group of non‐users. The strategy used for classification was *k*‐fold cross‐validation (*k* = 10), with one subject per class held out in each fold [25]. Significance was assessed via permutation testing with 100 permutations for MKL and 1000 for SVM and GPC. Also, the Support Vector Machine (SVM) and Gaussian Process Classifier (GPC) algorithms were performed with similar cross‐validation procedures and 1000 permutations. The SVM and GPC algorithms were also applied, both of which yield sparse discriminative information. Full model performance metrics and region‐wise contributions can be found in the Supplementary Results (Supporting Information).

In the pattern recognition analysis in favor of regression between the pattern of all voxels of each volunteer and the value of the psychometric assessments of each volunteer, was used the Gaussian Process Regression (GPR) algorithm [26]. In the GPR model, the concept of a Gaussian distribution extends to a probability distribution of functions assured by Bayes' rule to identify the distribution function that best matches the data or target values of regression. A regression analysis of the various psychometric assessments was performed only if the main scale showed significant differences between groups and in the scale which all subjects completed. The cross‐validation strategy used in this regression algorithm was “leave one subject out” and the *p*‐value was obtained through a permutation test with 1000 permutations.

## 4 Statistical Analysis

Group comparisons of psychometric scores were performed using GraphPad Prism version 10.0 (GraphPad Software, San Diego, CA, USA). Student's *t*‐tests for normally distributed variables and Mann–Whitney *U* tests for non‐normally distributed variables were conducted, as determined by the D'Agostino–Pearson omnibus normality test.

Multivariate pattern classification and regression analyses were conducted using machine learning algorithms: Multiple Kernel Learning (MKL), Support Vector Machine (SVM), and Gaussian Process Classification/Regression (GPC/GPR). Models were trained on whole‐brain fMRI data preprocessed in SPM12 (Wellcome Center for Human Neuroimaging, University College London, London, UK) and analyzed using the PRoNTo toolbox (version 3.0, University College London, London, UK), implemented in MATLAB 2016b (MathWorks Inc., Natick, MA, USA). Model performance was evaluated using 10‐fold cross‐validation, and statistical significance was assessed via permutation testing (*n* = 100 for MKL; *n* = 1000 for SVM and GPC/GPR). The threshold for statistical significance was α = 0.05. The area under the ROC curve (AUC) was computed to evaluate the discrimination performance of each classification model.

## 5 Results

### 5.1 Psychometric Assessments

**FIGURE 1:**
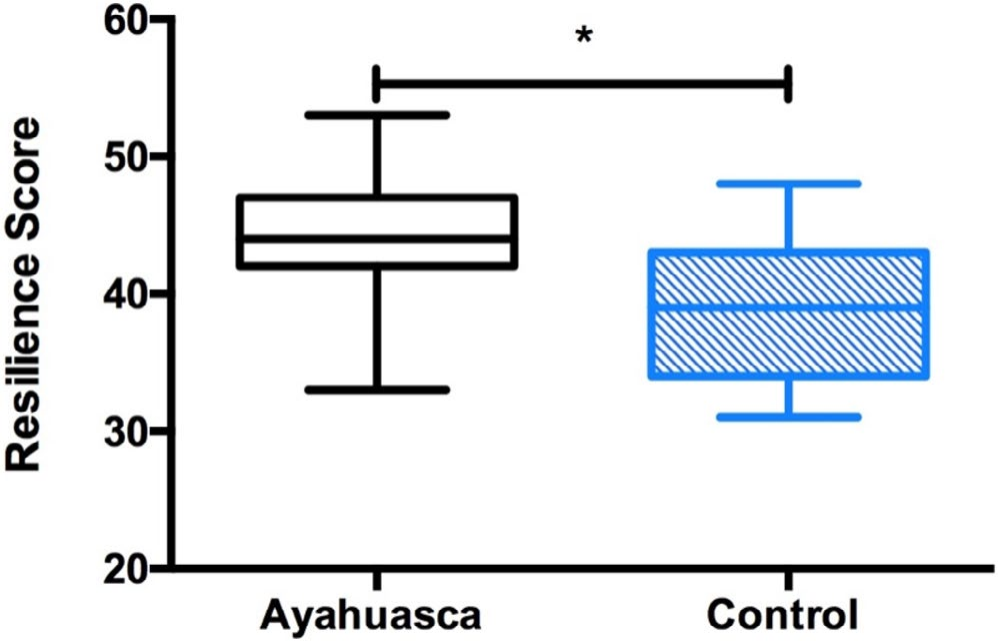
Boxplot of resilience measured by the ER‐89 scale in Ayahuasca group (black box) and control Group (blue box). *p*‐value = 0.01*.

The mean ER‐89 resilience score was higher for the Ayahuasca group (mean = 43.89; SD = 5.64) compared to the control group (mean = 39.05; SD = 5.34) (Figure 1). Meanwhile, the PANAS shows that there is no difference between groups in the positive effect (*p* = 0.72) and negative effect (*p* = 0.56). The STAI inventory shows that there is no difference in anxiety between groups, both in the anxiety trait (*p* = 0.30) and in the state anxiety (*p* = 0.17). The Beck Anxiety Inventory shows no difference between groups in symptoms of anxiety (*p* = 0.35). The Beck Depression Inventory shows no difference between groups in symptoms of depression (*p* = 0.69). Table 1 shows these statistics for both groups.

**TABLE 1:** Comparison of the scales of resilience, positive affect, and negative affect, state–trait anxiety, symptoms of anxiety, and depression between groups.

	Ayahuasca group		Control group		*p*
	Mean	SD	Mean	SD	
Resilience	43.89	5.64	39.05	5.34	0.01*
Positive affect	33.74	4.94	32.84	5.97	0.72
Negative affect	14.74	4.69	13.95	3.60	0.56
STAI – trait	31.11	8.48	33.56	5.20	0.30
STAI – state	27.79	6.43	30.89	6.90	0.17
BAI	2.68	2.52	2.00	1.91	0.35
BDI	5.37	4.72	4.74	4.13	0.69

*Note*: Independent two‐sample Student's *t*‐test between groups was applied in each scale. Statistical threshold: *a* = 0.05.

Abbreviations: BAI = Beck anxiety inventory; BDI = Beck depression inventory; SD = standard deviation; STAI = state–trait anxiety inventory.

### 5.2 Pattern Classification Models in fMRI Data

**FIGURE 2:**
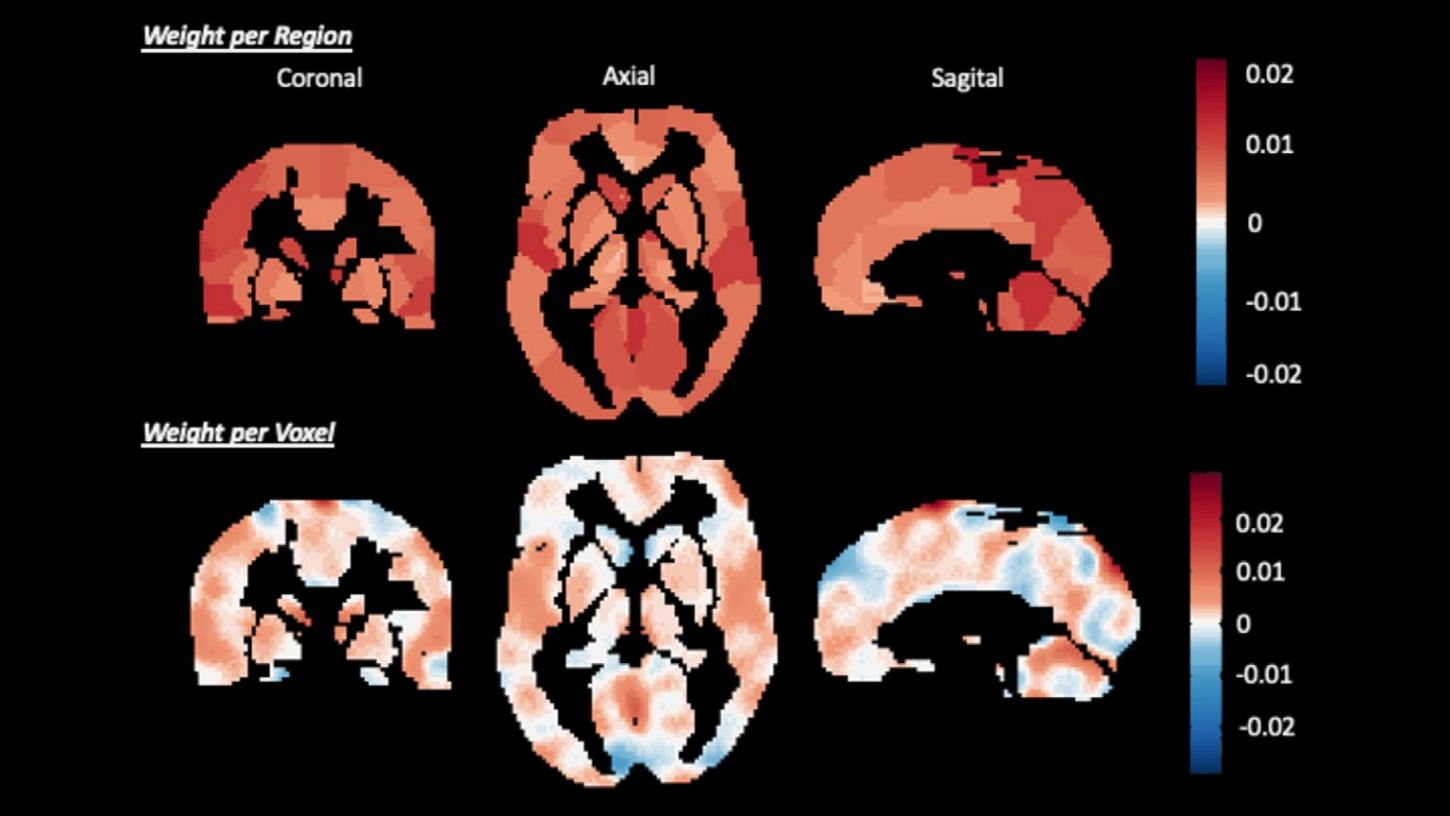
Weight map using the MKL algorithm with the AAL3 mask and *k*‐fold 10. This algorithm incorporates weights for each anatomical region and voxel, specified by a mask.

The MKL algorithm based on the AAL3 atlas and *k*‐fold cross‐validation (*k* = 10) was able to discriminate the classes of subjects, having a significant total accuracy of 75%, an accuracy in the class of users of 65% (*p* = 0.10), a significant accuracy in the non‐user class of 85%, and an area under the ROC (Receiver Operating Characteristic) curve of 0.68 (Table 2). The MKL weight map (Figure 2) highlights the most relevant regions for group classification. Table 3 presents 10 anatomical regions of the AAL3v1 atlas that most contributed to this discrimination model.

**TABLE 2:** Performance metrics to discriminate fMRI pattern activation between Ayahuasca and control group.

Models	Cross‐validation strategy	Total accuracy	Class 1 (Ayahuasca group)	Class 2 (control group)	ROC
MKL	*K*‐folds CV (*k* = 10)	75% (*p* = 0.005*)	65.00% (*p* = 0.10)	85% (*p* = 0.003*)	0.68
SVM	*K*‐folds CV (*k* = 10)	77.5% (*p* = 0.002*)	70.00% (*p* = 0.06)	85% (*p* = 0.002*)	0.82
GPC	*K*‐folds CV (*k* = 10)	77.5% (*p* = 0.003*)	70.00% (*p* = 0.06)	85% (*p* = 0.002*)	0.78

*Note*: The *p*‐values were obtained by permutation test (100 permutations for MKL algorithm and 1000 for SVM and GPC) using *K*‐folds cross‐validation on subject per class procedure (*k* = 10) and leaving one subject per class out (LOSPCO). Statistical threshold: *a* = 0.05.

Abbreviations: CV = cross‐validation; GPC = Gaussian process classification; MKL = multiple kernel learning; ROC = receiver operating characteristic; SVM = support vector machine.

**TABLE 3:** The 10 anatomical regions that contribute to discrimination in the MKL algorithm using the AAL3 map and *K*‐fold cross‐validation on the subject per class procedure (*k* = 10) validation between the Ayahuasca group and the control group, with their respective weights and voxel numbers.

Rank	Anatomical description	ROI label	Kernel weight (%)	ROI size (voxel)
1	Anteroventral nucleus thalamus, R	Thal_AV_R	2.13	23
2	Crus I of cerebellar hemisphere, R	Cerebellum_Crus1_R	2.01	90
3	Superior parietal gyrus, R	Parietal_Sup_R	1.77	1007
4	Anteroventral nucleus thalamus, L	Thal_AV_L	1.61	18
5	Supramarginal gyrus, L	SupraMarginal_L	1.55	1214
6	Crus I of cerebellar hemisphere, L	Cerebellum_Crus1_L	1.44	182
7	Paracentral lobule	Paracentral_Lobule_	1.41	776
8	Heschl's gyrus, L	Heschl_Left	1.36	222
9	Lobule VII of vermis	Vermis_7	1.28	31
10	Superior parietal gyrus, L	Parietal_Sup_L	1.23	1420

Abbreviations: L = left; *R* = right; ROI = region of interest.

### 5.3 Pattern Regression Models Between fMRI Data and Resilience Trait

The regression algorithms GPR from the pattern of all voxels of each volunteer were able to predict the value of the trait resilience scale of each volunteer in both groups, measured by the psychometric scale ER‐89, using two *k*‐fold cross‐validation strategies (*k* = 10 and *k* = 5). The GPR algorithm was able to correlate these different measures with a correlation of 0.69 (*R* ^2^ = 0.50) with an MSE of 35.72% (*p* = 0.15) and its normalized value of 1.13 (*p* = 0.09) (Table 4).

**TABLE 4:** Performance metrics to predictive function from fMRI pattern activation to resilience trait.

Models	Cross‐validation strategy	*r*	*R* ^2^	MSE	Normalized MSE
GPR	*k* = 10	0.69 (*p* = 0.002*)	0.50 (*p* = 0.05*)	35.72 (*p* = 0.15)	1.13 (*p* = 0.09)
	*k* = 5	0.56 (*p* = 0.005*)	0.32 (*p* = 0.04*)	37.89 (*p* = 0.21)	1.08 (*p* = 0.19)

*Note*: The *p*‐values were obtained by permutation test (1000 permutations) using 10‐fold cross‐validation procedure.

Abbreviations: GPR = Gaussian process regression; MSE = mean square error.

## 6 Discussion

The main goal of the present study was to evaluate the long‐term effect of the use of Ayahuasca through psychometric scales and fMRI data that assess emotional processing in the brain using artificial intelligence analysis techniques that allow detecting subtle effects at an individual level. Our psychometric results showed that the trait of resilience among the Ayahuasca group was greater than the control group, whereas there were no differences in symptoms of anxiety and depression or positive affect and negative affect between groups. Furthermore, using a pattern regression model, the value of the resilience scale of each subject could be inferred from their brain patterns. In addition, the pattern classification model of the fMRI data was also able to discriminate which volunteers belonged to each group.

The Ayahuasca group exhibited a higher resilience trait compared to the control. The resilience trait is related to openness, that is a personality trait characterized by a willingness to engage with new experiences, ideas, and unconventional values, reflecting curiosity and a broad range of interests [27]. Previous studies have shown openness is enhanced by Ayahuasca ingestion in both the acute phase [2] and the long‐term phase [28]. Another study evaluates long‐term effects observed through psychometric scales and cognitive tests, confirming that Ayahuasca does not cause mental health deterioration or cognitive impairment [29]. Currently, acute changes in behavior and cognitive states have been widely observed due to Ayahuasca ingestion [5, 6, 30], including showing differences in executive function between experienced and inexperienced individuals in the use of Ayahuasca [31].

Moreover, the present results highlight the relationship between brain activity and the trait of resilience. The machine learning algorithm successfully predicted resilience scores based on the brain activity of volunteers from both groups, demonstrating a neural signature of resilience in an experiment with emotional response stimuli. Coupled with the observed differences in resilience between the groups, this may indicate brain plasticity and, consequently, behavioral changes due to the chronic use of Ayahuasca.

In this analysis, the MKL algorithm incorporates during the learning of a discriminant function information from anatomical regions and from the hierarchy of individual voxels. In the classification model, the largest contributions to classify Ayahuasca from the control group were distributed on the parietal region, medial frontal cortex, limbic regions (including amygdala, insula and anterior cingulate cortex), occipital cortex, and basal ganglia (including thalamic and raphe nuclei). It is noteworthy that, even with a more concentrated weight distribution by these anatomical regions, all regions effectively participated in the decision function. The MKL algorithm, based on the pattern of brain activation during aversive stimuli processing, selected the regions that contributed the most to the discriminate function; therefore, weight maps do not represent exactly the brain activation in that specific region during the task. Thus, while they are useful for obtaining information about relative importance, they do not provide insight into the underlying mechanisms. Although there is a correlation between brain activity and weights for a decision function, the present results only indicate that the activity of this region was important for the decision‐making process [32]. For example, the parietal region is related to sensorimotor integration, in controlling spatial attention to emotional information [33] and in emotion regulation [34]. Also, it is observed that the experienced meditators evoke parietal regions during emotional protocols [35, 36].

The amygdala is considered a hub in the mediation of emotional and social behavior [37]. A review by Bombardi [38] showed consistent data on the distribution and functional role of the 5‐HT2A receptor in the amygdala, a fundamental receptor in the activity of hallucinogenic and psychedelic compounds. Riba et al. [6] in the first study via SPECT observed that the ingestion of Ayahuasca promotes an acute increase in blood flow in areas related to the emotional network, such as: amygdala; anterior insula; inferior frontal gyrus; frontal medial; anterior cingulate; parahippocampal gyrus. In a previous study including the same Ayahuasca participants and the same fMRI paradigm to evaluate the acute effects of Ayahuasca, the amygdala emotional reactivity was down regulated, and these participants reported reduced anxiety symptoms after intake [9]. Thus, the considerable contribution of the amygdala to discrimination between groups is interesting, as it participates both in the emotional circuitry evoked by the task and because studies show its high biochemical influence.

The medial frontal cortex is related to executive processes and highly complex cognitive processing, being a classic region in emotional processes [39]. Recently, Sperl et al. [40] performed a face processing experiment using fMRI and EEG techniques simultaneously, which promoted the understanding of the top‐down circuitry relationship between the medial frontal cortex and the amygdala. It is noteworthy that in the SPECT experiments with Ayahuasca [6, 41], both the amygdala and the medial frontal cortex had an increase in blood flow after the use of Ayahuasca, demonstrating a high interaction of these regions with this compound.

The visual cortex is a brain region responsible for three‐dimensional processing and is sensitive to the perception of movement of the subject and the environment around them [42], receiving its sensory inputs through the thalamus. The anterior nuclei of the thalamus and other nuclei from the medline are considered the “limbic” thalamic nuclei [43]; they connect cortical and subcortical structures like the amygdala and the nucleus accumbens to the medial prefrontal cortex and other cortical regions. Although a previous study showed increased visual activity after Ayahuasca ingestion in experienced volunteers [44], the volunteers in this study did not consume Ayahuasca. Therefore, the greater importance of visual regions for discrimination function may be related to the lack of processing bias of emotional stimuli in some of the groups. The most accentuated contribution of both the basal ganglia (including thalamus nuclei) and the occipital region may be linked to the sensitivity of these regions to facial expressions of disgust and fear and may be related to the perception and regulation of emotion in aversive social interaction situations, since the literature points to these structures as having an integration with the emotional network [45, 46].

## 7 Limitations

This study had a small sample of volunteers due to the highly complex method of physiological measurement and the select criteria for screening volunteers in order to have a homogeneous group. In particular, only male participants were included, which limits the generalizability of our findings to female populations. A possible consequence of this limitation could be the absence of observation of effects in univariate analyses by GLM between groups. Therefore, further studies are needed to understand the short‐and long‐term effects of Ayahuasca on the body. For a large group, it would be possible to test different cross‐validation strategies, and greater extrapolation of results to larger populations should be considered. Future studies with control criteria and large samples should also allow the exploration of the use of Ayahuasca in specific regions of the brain, with emphasis on key regions in emotional regulation, such as the amygdala, insula, and prefrontal cortex.

## 8 Conclusion

This study uses fMRI to investigate the long‐term effects of using Ayahuasca. Our findings might indicate that experienced subjects who make regular use of Ayahuasca have different emotional brain reactivity than the control group, even when they are not directly under Ayahuasca's influence. We observed that it is possible to decode the differences between groups, in addition to the observation of long‐term behavioral effects related to resilience. Both results are related to changes in emotional reactivity in the brain. A possible explanation is that the chronic use of Ayahuasca promotes a modulation in brain activation to aversive stimuli, a pattern associated with resilience which could be indirectly involved in the emotion regulation process, altering the defensive response to aversive stimuli.

## Conflicts of Interest

The authors declare no conflicts of interest.

## Supporting information


**Data S1:** Supporting Information.
